# The impact of multiple factors on medical students' test anxiety: the mediating role of psychological resilience

**DOI:** 10.3389/fpsyg.2026.1802740

**Published:** 2026-05-21

**Authors:** Jing Chen, Fei Chen, Shuang Qiu

**Affiliations:** 1 The Fifth Clinical College, Hubei University of Medicine, Shiyan, China; 2 Hubei University of Medicine, Shiyan, China

**Keywords:** academic stress, perfectionism, psychological resilience, social support, test anxiety

## Abstract

**Background:**

This study examined the influence of academic stress, social support, and perfectionism on test anxiety among medical students, as well as the mediating role of psychological resilience. Drawing on Control-Value Theory and Resilience Theory, the study explored how internal protective resources respond to academic, environmental, and personality-related factors.

**Methods:**

A structured questionnaire was administered to medical students from three medical universities in China through a convenience sampling approach. A total of 1,067 valid responses were collected. SPSS 26.0 was utilized to perform statistical analyses and explore the associations among the variables.

**Result:**

The results showed that academic stress and maladaptive perfectionism were significantly positively related to test anxiety, whereas social support and adaptive perfectionism were significantly negatively related to it. Psychological resilience was found to mediate the relationships between the predictor variables and test anxiety. Specifically, the mediation effect accounted for 18.889% (academic stress), 56% (social support), 30.588% (adaptive perfectionism), and 12.658% (maladaptive perfectionism) of the total effects, respectively.

**Conclusion:**

The findings highlighted the central role of psychological resilience in mitigating the negative impact of academic stress and maladaptive perfectionism on test anxiety, while also strengthening the positive influence of social support and adaptive perfectionism. These outcomes suggested that strategies designed to cultivate resilience may help alleviate test anxiety and enhance both psychological health and academic performance among medical students.

## 1 Introduction

Medical students face a demanding curriculum and intensive practical requirements, leading to considerable academic stress (AS) during their university years (Sherina et al., 2004). Individuals pursuing studies in the field of medicine often face intense academic and emotional demands, which places them at elevated risk for psychological strain. This vulnerability is reflected in the frequent occurrence of mental health concerns, including persistent low mood, internal unrest, and disrupted sleeping habits—issues less commonly reported among students from other majors (Dyrbye et al., 2006; Peng et al., 2023). In China, undergraduate medical education is heavily oriented toward memorization, making examinations the primary method for evaluating students' academic performance. Moreover, to boost their career competitiveness, many medical students choose to take additional high-stakes exams, such as the postgraduate entrance examination and the National Medical Licensing Examination. These examinations, known for their extensive scope and intense competitiveness, often place considerable psychological strain on medical students, thereby undermining their emotional wellbeing (Chen et al., 2022; Wang, 2022). The dominance of examination-based assessment practices is widely regarded as a major influence underlying the prevalence of test anxiety (TA) among medical students (Abdel Wahed and Hassan, 2017).

Test anxiety is a psychological state characterized by tension and concern specifically related to examinations and performance outcomes, and is generally composed of two elements: cognitive worry and emotional arousal (Rana and Mahmood, 2010; Sarason and Sarason, 1990). Scholars have found that a mild level of TA can enhance academic performance, whereas excessive TA tends to impair it (Chapell et al., 2005; Khalaila, 2015). Several studies found that elevated TA is associated with a range of adverse academic consequences, including academic burnout, poor performance, and academic procrastination (Bolbolian et al., 2021; Cassady and Johnson, 2002; Fisher et al., 2024). Factors influencing TA are generally categorized into three dimensions. The first is personal characteristics, such as perfectionism (Abdollahi et al., 2018), self-efficacy (Nie et al., 2011), and psychological resilience (Alazemi et al., 2023). The second is environmental factors, such as family functioning (Chen et al., 2023) and social support (Yildirim et al., 2008). The third is academic factors, including AS (Zheng et al., 2023) and goal orientation (Putwain and Daniels, 2010).

Guided by Control-Value Theory (Pekrun, 2006) and Resilience Theory (Masten, 2001), the current study integrates the three dimensions—academic, environmental, and individual—to explore how AS, social support (SS), and perfectionism contribute to TA among medical students. It also examines in depth the mediating role of psychological resilience (PR) in this process. While existing studies have provided limited insight into the factors influencing TA in medical students, this research seeks to address that gap and deepen both academic and educational understanding of this issue. In practical terms, the overwhelming load of examinations imposes considerable mental strain on medical students. Therefore, the study's findings could provide both theoretical guidance and practical strategies aimed at reducing academic stress, enhancing psychological wellbeing, and supporting the academic development of medical students.

## 2 Literature review

### 2.1 Theoretical basis

The Control-Value Theory (Pekrun, 2006) posits that emotional experiences, such as TA, are primarily influenced by individuals' perceptions of control and value. Perceived control reflects how strongly a person feels capable of regulating and managing academic demands through their own efforts. When students perceive themselves as lacking the necessary abilities or resources to cope with academic challenges, their sense of control diminishes. Perceived value refers to the subjective importance or significance attached to the outcome of a task (Perez et al., 2019). When students place high importance on exam results, their sense of value increases (Roick and Ringeisen, 2017). This theoretical model suggests that students are more prone to emotional discomfort, including nervousness, stress, or anxiety, during exams when they feel powerless but place high importance on the results (Pekrun et al., 2007). This theoretical framework offers crucial support for exploring the mechanisms through which multiple factors contribute to TA in medical students.

Resilience Theory (Masten, 2001) emphasizes the ability of individuals to recover or preserve stable psychological functioning when facing challenges or stressful circumstances. Masten (2001) emphasized that PR is not an extraordinary trait but rather an adaptive process built upon commonly accessible resources such as positive cognitive coping strategies and SS. In the context of AS and perfectionism, PR serves as a vital protective resource that can enhance individuals' sense of control and coping efficacy, thereby mitigating the negative effects of AS and maladaptive perfectionism (Masten, 2018). Specifically, PR enables students to respond to stressful situations more proactively by enhancing the flexibility and adaptability of their coping strategies, which in turn helps reduce levels of TA (Rademacher et al., 2023). Moreover, resilience may help students reframe the perceived value of exam outcomes in a more balanced manner, thereby alleviating excessive anxiety caused by maladaptive perfectionism (MP).

Therefore, this study integrates Control-Value Theory and Resilience Theory to uncover the mechanisms through which multiple factors influence TA among medical students. On one hand, Control-Value Theory explains how AS, SS, and perfectionism affect TA through the modulation of perceived control and value. On the other hand, Resilience Theory underscores the role of personal adaptability in countering both outside stress and perfectionistic self-demands through fostering a stronger sense of agency and reshaping the significance attached to academic performance. Psychological resilience acts both as a direct protective factor for TA and as an essential mediator linking AS, SS, and perfectionism to TA outcomes. By combining these two theories, this study seeks to examine the complex mechanisms behind the factors influencing TA and to offer a theoretical foundation and empirical evidence for addressing TA in medical undergraduates.

### 2.2 Academic stress and test anxiety

Academic stress refers to the negative emotional and alert responses that arise when students perceive academic demands to exceed their available coping resources (Baum, 1990; Wilks, 2008). Evidence from studies conducted in regions such as Thailand, Pakistan, and the U.S. suggests that those pursuing medical education are more susceptible to intense scholastic pressure than students enrolled in non-medical majors (Mosley et al., 1994; Saipanish, 2003; Waqas et al., 2015). In China, factors such as prolonged academic training, mandatory clinical rotations, and the burden of career-related decision-making contribute to a notably elevated AS level among medical students (Mao et al., 2019). An overwhelming AS threatens medical students' overall health, potentially leading to both physiological strain and psychological distress. As demonstrated in extant literature, elevated stress levels have been associated with the onset of sleep dysfunction (Almojali et al., 2017), alterations in daily eating habits (Alduraywish et al., 2023), and can contribute to the emergence of depression, as well as a spectrum of emotional disturbances and physiological complications (Romo-Nava et al., 2016). Moreover, excessive AS negatively impacts students' academic performance, reducing their academic engagement and leading to poorer academic outcomes (Alotaibi et al., 2020; Sinval et al., 2025).

According to stress-coping theory (Lazarus, 1993) and the stress-vulnerability model (Zubin and Spring, 1977), individuals exposed to prolonged AS are more likely to develop cognitive vulnerabilities, making them more susceptible to psychological problems, including heightened levels of TA. Current studies have demonstrated that AS significantly contributes to increased TA among university students (Jody and Andrews, 2013; Zheng et al., 2023). For instance, Zheng et al. (2023), using a questionnaire survey, found that when Chinese university students perceived high AS, their emotional control self-efficacy decreased, leading to elevated TA. This relationship has also been confirmed in medical students. Zhang et al. (2022) found that rising AS not only intensified TA symptoms among medical students but also contributed to a stronger sense of helplessness during their studies, further increasing the likelihood of developing depressive symptoms. In summary, AS significantly contributes to the increase of TA among medical students.

### 2.3 Social support and test anxiety

Social support refers to an individual's perception of receiving care, respect, and assistance from people within their social environment (Taylor, 2011). As a vital element of mutual social relationships, it includes emotional, informational, and tangible assistance, all of which play an essential role in enhancing both psychological and physical health (Gariepy et al., 2016; Taylor, 2011). Social support is typically categorized into four types: emotional support, including expressions of care, empathy, affection, and trust; instrumental support, involving tangible assistance such as goods or services; informational support, referring to problem-solving advice or guidance; and appraisal support, involving feedback that aids in self-evaluation (Langford et al., 1997). Previous research suggest that adequate SS is crucial in safeguarding the psychological wellbeing of medical students, a factor evidenced by the alleviation of depressive and anxious symptoms (Thompson et al., 2016; Yin et al., 2021). Moreover, such supportive environments are instrumental in advancing their educational performance and future professional success. For example, sufficient SS can enhance medical students' academic self-perception (Yamada et al., 2014), reduce academic burnout (Zhang et al., 2021), and foster positive professional attitudes (Yang et al., 2022).

According to Control-Value Theory (Pekrun, 2006) and the stress-buffering model (Cohen and Wills, 1985), when individuals face stress-inducing events such as examinations, SS helps mitigate the adverse effects of these stressors on psychological and physical health by enhancing one's sense of control. Therefore, SS plays a stabilizing role by mitigating TA and safeguarding the mental health of medical students (Almutairi et al., 2024; Kassaw et al., 2024; Tsegay et al., 2019). Almutairi et al. (2024), through survey-based research, observed that Filipino students in medical programs who lacked adequate SS tended to suffer from more intense forms of TA. Similarly, Tsegay et al. (2019) reported that low to moderate levels of SS positively predicted TA among medical students, with students receiving low levels of support being three times as prone to high TA as their peers who had strong support systems. These evidence points to the crucial function of interpersonal support networks in easing exam-related psychological distress within the medical student population.

### 2.4 Perfectionism and test anxiety

Defined as a cognitive-behavioral disposition, perfectionism involves relentless self-imposed demands, an uncompromising drive for flawlessness, and a critical attitude toward one's own shortcomings (Stoeber and Otto, 2006). Perfectionism frequently reveals itself through intense self-criticism, uncertainty about one's achievements, sensitivity to parental demands, and an excessive emphasis on precision, orderliness, and organization (Frost et al., 1990). Hamachek (1978) was the first to distinguish between adaptive perfectionism (AP) and MP. The former refers to individuals who pursue high standards while still experiencing satisfaction and a sense of achievement upon meeting those standards. In contrast, the latter involves setting unrealistically high expectations with little tolerance for error. These two forms of perfectionism have markedly different effects on individuals. Adaptive perfectionism is generally beneficial, promoting personal growth and enhancing students' academic engagement and performance (Closson and Boutilier, 2017; Osenk et al., 2020; Park et al., 2020). Maladaptive perfectionism, in contrast, tends to trigger excessive self-critical thoughts and emotional strain, and has been associated with various mental health issues such as disordered eating, insomnia, depression, and suicidal thoughts (Bieling et al., 2004; Hewitt et al., 1997; Lombardo et al., 2013; Vincent and Walker, 2000; Wang and Li, 2017).

According to Control-Value Theory (Pekrun, 2006), medical students exhibiting strong maladaptive perfectionistic traits are inclined to perceive examinations as threatening and to underestimate their ability to cope due to excessively high self-imposed standards. By comparison, those characterized by adaptive perfectionistic traits often demonstrate a balanced view of their scholastic capabilities and are less emotionally affected by test performance outcomes (Madigan, 2019). As a result, elevated MP tends to correspond with heightened TA, whereas higher AP appears to be related to lower levels of TA among medical students (Ahmadi et al., 2025; Burcaş and Cretu, 2021; Eum and Rice, 2011; Nascimento da Silva et al., 2025; Weiner and Carton, 2012). However, findings on the effects of the two types of perfectionism on TA remain inconsistent. For example, Eum and Rice (2011) found that MP significantly predicted higher TA, while AP had no direct association with it. According to (Burcaş and Cretu 2021), findings from their meta-analysis revealed that individuals exhibiting MP were considerably more susceptible to TA, but only a small and non-significant positive correlation with AP. Ahmadi et al. (2025) found that students with moderate levels of perfectionistic tendencies showed lower TA, whereas more extreme perfectionistic tendencies were associated with higher perceived threat. Notably, Wan et al. (2024) reported that, among junior high school students in China, AP was a negative predictor of TA, whereas MP showed no significant association. They suggested that this might be due to the high frequency of examinations in East Asian educational contexts, which increases students' exposure to test-related setbacks and may cause those with maladaptive perfectionist tendencies to internalize failure and engage in self-blame. In summary, AP appears to alleviate TA among medical students, whereas MP tends to intensify it.

### 2.5 The potential mediation role of psychological resilience

Psychological resilience, as an essential personal trait activated during adversity, significantly influences the strategies individuals use to manage challenging circumstances (Connor and Davidson, 2003). According to Resilience Theory, PR plays two crucial roles: it protects individuals by mitigating the effects of negative variables and preventing further negative consequences, while also boosting self-esteem and self-efficacy to encourage positive, adaptive responses (Rutter, 1985). Given these functions, PR plays a critical role in the mental health of medical students. Studies have found that when PR is lower among medical students, burnout levels tend to be higher (Forycka et al., 2022) and a greater vulnerability to the onset of depressive emotional states (Zhao et al., 2022, 2021). Other researches have confirmed that PR significantly reduces TA (Hayat et al., 2021; Li et al., 2026; Liu et al., 2021; Trigueros et al., 2020). Li et al. (2026) found that mindfulness was associated with lower TA among high school students through higher PR. This finding also applies to medical students. Hayat et al. (2021) highlighted that medical students' self-efficacy significantly predicts their level of PR, which in turn alleviates TA.

Resilience Theory posits that levels of PR are primarily influenced by risk factors and protective factors (Olsson et al., 2003). Risk factors refer to challenging life experiences that may trigger problems or prolong difficulties, such as illness, emotional distress, stress, and MP (Barbarić et al., 2020; Klibert et al., 2014; Rutter, 1985). Protective factors refer to contextual or external resources that support individuals in resisting and balancing the effects of adversity (Olsson et al., 2003; Rutter, 1985). These include SS and the school climate (Kang et al., 2024; Mateos et al., 2020). Therefore, AS, SS, and perfectionism may influence medical students' TA through the mediating role of PR.

Current studies have confirmed that AS can reduce medical students' PR, negatively affecting their mental health and learning efficiency (Berdida, 2023; Yang et al., 2025). For example, Berdida (2023) found that when AS is excessive among nursing students, their PR declines, along with a reduction in their autonomous learning capacity. However, PR may also play a buffering role, making medical students less susceptible to the negative effects of AS. Ho et al. (2023) found that among Vietnamese adolescents, higher PR reduced the likelihood of developing depressive symptoms under AS. Therefore, PR could serve as an intermediary mechanism linking AS to TA.

Multiple studies have shown that when medical students perceive sufficient SS, their levels of PR also tend to increase (Ho et al., 2025; Liu and Cao, 2022; Naz et al., 2024; Thompson et al., 2016). For instance, Liu and Cao (2022) found that among Chinese medical students, SS enhanced PR, thereby helping to reduce academic burnout. Similarly, Naz et al. (2024), through quantitative analysis, found that SS from family and friends improved PR among nursing students, enabling them to adopt adaptive coping strategies and maintain their mental health. These results indicate that PR may mediate the relationship between SS and TA among medical students.

Research evidence also suggests that MP in medical students is often linked to reduced PR, while AP is positively correlation with PR (Jiao et al., 2025; Klibert et al., 2014; Sheppard and Hicks, 2017; Wan et al., 2023). As shown in the study by Wan et al. (2023), adaptive perfectionistic tendencies among nursing students in China strengthened their belief in their own abilities, which subsequently promoted PR and boosted learning motivation, whereas MP had the opposite effect in this chain mediation model. According to Klibert et al. (2014), students who internalized socially imposed standards—a manifestation of MP—tended to show weaker PR when facing adversity and were more susceptible to depressive emotional states. Sheppard and Hicks (2017) also observed that MP reduced PR and increased psychological distress among university students. In summary, MP reduces PR and increases TA among medical students, while AP enhances PR and thereby helps alleviate TA.

### 2.6 The current study

**Figure 1:**
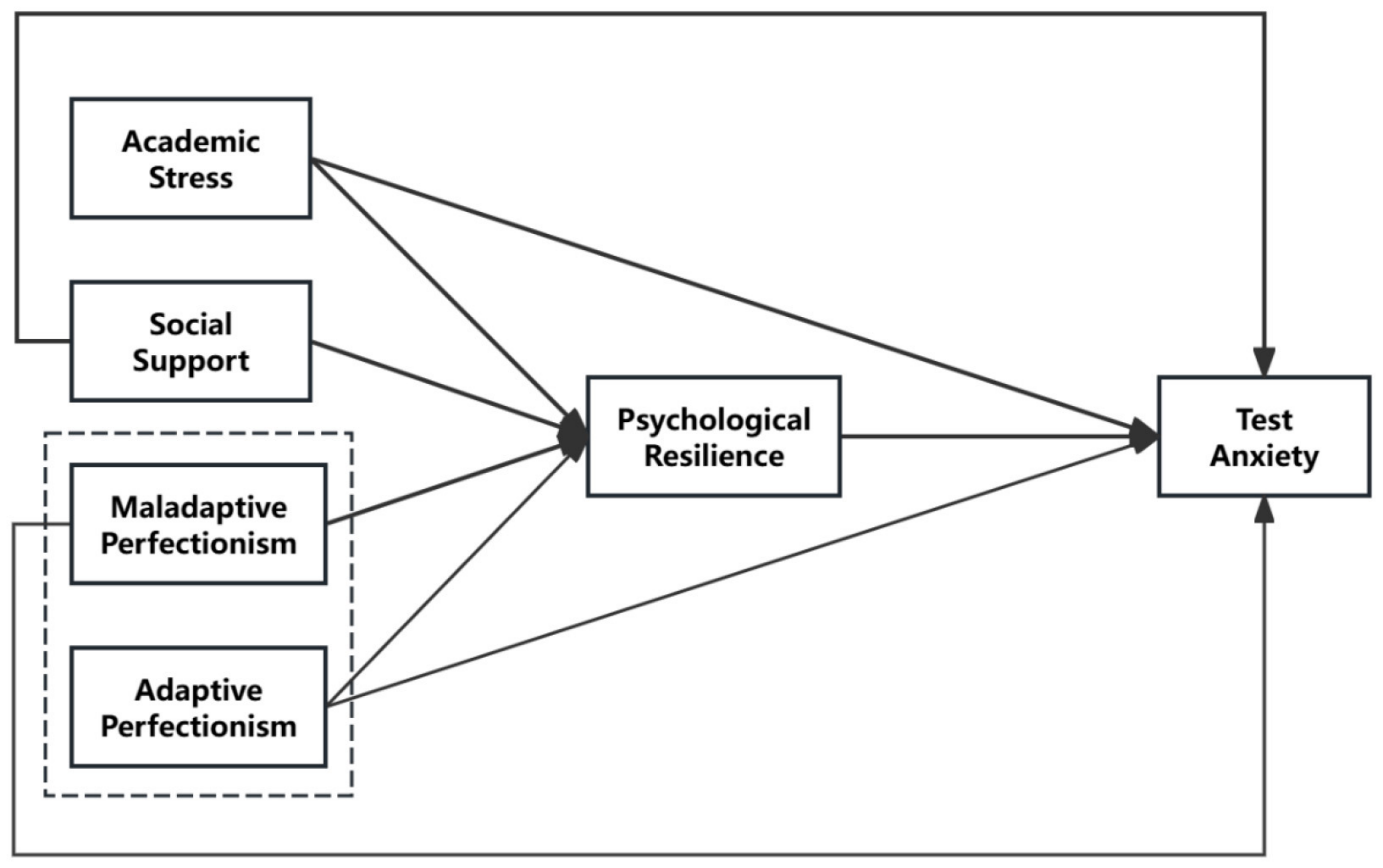
The research model.

Based on Control-Value Theory and Resilience Theory, this study selects AS, SS, and perfectionism as independent variables to examine their effects on TA among medical students. Psychological resilience is incorporated into the model as a mediator to emphasize its pivotal function in protecting the psychological stability of medical students, as revealed through mediation analysis. Grounded in the preceding theoretical and empirical insights, this study proposes a series of hypotheses as outlined below, and the research model is illustrated in Figure 1.

H1: AS has a significant positive effect on TA among medical students.

H2: SS has a significant negative effect on TA among medical students.

H3: MP has a significant positive effect on TA among medical students.

H4: AP has a significant negative effect on TA among medical students.

H5: PR mediates the relationship between AS and TA.

H6: PR mediates the relationship between SS and TA.

H7: PR mediates the relationship between MP and TA.

H8: PR mediates the relationship between AP and TA.

## 3 Method

### 3.1 Data collection

During the period from December 2024 to January 2025, data were gathered from students at three medical universities in China using a convenience sampling approach and structured questionnaire distribution. During this period, medical students were undergoing their final examinations, making it an appropriate time to investigate issues related to TA. An online questionnaire was distributed to participants using Wenjuanxing (https://www.wjx.cn/), allowing students to complete it using mobile phones, computers, or other devices. The survey was conducted with the approval of the faculties responsible for the medical programs at the participating universities. Prior to participation, students received an informed consent form detailing the study's purpose, the voluntary nature of their involvement, the confidentiality of their responses, and their right to withdraw at any time without consequences. Only those who consented proceeded with the questionnaire. To avoid any perception of coercion, participation was entirely voluntary, and instructors were not involved in students' responses and had no access to any individual data. After students were informed about the purpose and procedures of the study, instructors at each university assisted the research team in distributing the survey invitation to eligible students. To ensure ease of access, the questionnaire was distributed in two formats: a scannable QR code and an online survey link. A total of 1,111 questionnaires were collected. After data screening, 34 questionnaires were excluded due to incompleteness or careless responses, resulting in 1,067 valid responses.

The demographic characteristics of the sample are presented in Table 1. The gender distribution among the participants was fairly balanced, with females comprising 52.296%, slightly outnumbering the male participants. Participants aged 18 to 22 comprised the largest age group, representing 84.536% of the sample. The distribution of participants across academic years was fairly balanced, with third-year students making up the largest group (29.709%) and fifth-year students the smallest (9.560%). Regarding academic disciplines, most respondents majored in clinical medicine (31.303%), followed by dentistry (14.620%), while the remaining medical fields were more uniformly represented.

**Table 1:** Frequency analysis of demographic variables.

Variable	Classification	Frequency	Percentage
Gender	Male	509	47.704%
	Female	558	52.296%
Age	Under 18 years old	31	2.905%
	18~20 years old	384	35.989%
	20~22 years old	518	48.547%
	Over 22 years old	134	12.559%
Grade	First year	182	17.057%
	Second year	229	21.462%
	Third year	317	29.709%
	Fourth year	237	22.212%
	Fifth year	102	9.560%
Major	Clinical medicine	334	31.303%
	Nursing	92	8.622%
	Pharmacy	63	5.905%
	Dentistry	156	14.620%
	Preventive medicine	91	8.529%
	Chinese medicine	81	7.591%
	Medical technology	129	12.090%
	Others	121	11.340%

### 3.2 Measures

The survey instrument employed in this study was divided into two sections. The initial section gathered general demographic details from participants, including gender, age, and academic year. In the second phase, Chinese versions of widely recognized measurement scales were applied to assess the five theoretical constructs explored in this research. To maintain linguistic precision and cultural relevance, a translation-back translation method was used, involving two groups of scholars fluent in both English and Chinese (Brislin, 1980). The research team carefully examined any inconsistencies between the original and back-translated versions, discussing and resolving them to maintain both semantic accuracy and conceptual alignment. In addition, minor wording adjustments were made where necessary to improve clarity and appropriateness in the context of Chinese medical students, while preserving the original meaning of the items. The reliability and validity of the translated instruments were further supported by acceptable Cronbach's alpha coefficients and confirmatory factor analysis (CFA) results in the present sample.

Test anxiety was measured using the Test Anxiety Scale (TAS) developed by Sarason (1978). The Chinese version of the scale has demonstrated good internal and external consistency in previous research (Liu et al., 2021). The TAS comprises one dimension and includes 37 items, of which items 2, 15, 26, 27, 29, and 33 are reverse scored. The scale adopts a binary scoring method (“yes” = 1 point, “no” = 0 points). Total scores between 0 and 11 indicate low TA, 12–19 indicate moderate TA, and 20 to 37 indicate high TA. In the current study, the TAS had a Cronbach's alpha of 0.764. CFA yielded the following fit indices: χ^2^/*df* = 3.184, RMSEA = 0.045, GFI = 0.885, AGFI = 0.872, IFI = 0.706, CFI = 0.704, and TLI = 0.686.

Academic stress was assessed using the Perception of Academic Stress Scale (PAS) developed by Gabriel (2015). The scale has shown good internal consistency in prior studies (Tin, 2024). The instrument evaluates stress across four key domains: performance-related pressure (five items), academic workload (four items), self-appraisal in learning contexts (four items), and time-related challenges (five items), comprising 18 items in total, five of which are reverse scored. Participants responded using a five-point Likert scale (1 = strongly disagree, 5 = strongly agree), where elevated scores signify more intense AS. In this study, the PAS demonstrated a Cronbach's alpha of 0.749, KMO = 0.848, and Bartlett's test was significant (*P* < 0.001), confirming good internal consistency. CFA results showed an acceptable model fit: χ^2^/*df* = 7.144, RMSEA = 0.076, GFI = 0.906, AGFI = 0.876, IFI = 0.728, CFI = 0.726, and TLI = 0.675.

Social support was measured using the Multidimensional Scale of Perceived Social Support (MSPSS) developed by Zimet et al. (1988). The reliability of the Chinese version has been well-documented in previous studies (Guan et al., 2015). The MSPSS contains 12 items and measures three sources of support: family, friends, and significant others, with four items per subscale. Participants responded using a seven-point Likert scale (1 = strongly disagree to 7 = strongly agree). In this study, the MSPSS yielded a Cronbach's alpha of 0.896, indicating high internal consistency. CFA results were as follows: χ^2^/*df* = 3.527, RMSEA = 0.049, GFI = 0.973, AGFI = 0.958, IFI = 0.973, CFI = 0.973, and TLI = 0.966.

Perfectionism was measured using the Short Almost Perfect Scale (SAPS) developed by Rice et al. (2013). The Chinese version of the scale has shown good reliability and validity in previous research Fong and Cai (2019). The SAPS contains eight items measuring two dimensions: standards (four items), reflecting the positive pursuit of high-performance standards, and discrepancy (four items), assessing excessive concern over the gap between expectations and actual performance. A five-point Likert scale was used, ranging from 1 (strongly disagree) to 5 (strongly agree). In this study, the standards subscale had a Cronbach's alpha of 0.741, indicating good consistency. CFA results for this subscale were: χ^2^/*df* = 6.662, RMSEA = 0.073, GFI = 0.994, AGFI = 0.968, IFI = 0.987, CFI = 0.987, and TLI = 0.961. The discrepancy subscale yielded a Cronbach's alpha of 0.726. CFA results for this dimension were: χ^2^/*df* = 5.006, RMSEA = 0.061, GFI = 0.995, AGFI = 0.977, IFI = 0.990, CFI = 0.990, and TLI = 0.970.

Psychological resilience was assessed using the 10-item version of the Connor-Davidson Resilience Scale (CD-RISC-10) developed by Campbell-Sills and Stein (2007). The Chinese version has demonstrated strong internal consistency (Cui and Chi, 2021; Niu et al., 2025). This unidimensional scale evaluates an individual's ability to cope with adversity, including stress, change, illness, and failure, through problem-solving, critical thinking, emotional regulation, and perseverance. Participants rate each item using a five-point Likert scale ranging from 0 (not true at all) to 4 (true nearly all the time), with higher scores reflecting stronger PR. In this study, the CD-RISC-10 showed a Cronbach's alpha of 0.859, confirming strong consistency. CFA results indicated good model fit: χ^2^/*df* = 4.452, RMSEA = 0.057, GFI = 0.971, AGFI = 0.955, IFI = 0.962, CFI = 0.962, and TLI = 0.951.

### 3.3 Statistical analysis

The data collected in this study were processed using SPSS version 26.0. The data analysis covered four core components. First, descriptive statistics were calculated, using kurtosis (*K*), skewness (*S*), means, and standard deviations (*M* ± SD) for continuous variables and frequencies for categorical variables. Pearson correlation analysis was employed to examine the associations between variables. Second, a common method bias analysis was conducted for the scales used in this study. Third, AMOS 26.0 was used to assess the model's goodness-of-fit indices. Fourth, after standardizing the relevant variables, mediation analysis was performed using the Bootstrap method in PROCESS macro 4.2 for SPSS. Specifically, Model 4 was selected for simple mediation analysis, with AS (*X*1), SS (*X*2), and perfectionism (*X*3) as independent variables, PR (*M*) as the mediating variable, and TA (*Y*) as the dependent variable. Demographic factors such as age and major were included as covariates. The significance of the mediating effect was tested using bias-corrected 95% confidence intervals (CI) based on 5,000 bootstrap samples (Mallinckrodt et al., 2006). If the bias-corrected 95% CI did not include zero, the mediating effect was considered statistically significant at the α = 0.05 level (Mackinnon et al., 2004).

## 4 Results

### 4.1 Descriptive statistics

Table 2 presents the results for *K, S, M*, SD, and Pearson correlation analysis for the variables examined in this study. The *K*-values for all scales were below three, indicating that the data fall within an acceptable range of normal distribution. The mean score for TA was 18.400, suggesting that participants, on average, experienced a moderate level of TA, though it remained relatively high. Significant positive correlations were found between AS (*r* = 0.511, *p* < 0.001) and MP (*r* = 0.321, *p* < 0.001) with TA. In contrast, SS (*r* = −0.322, *p* < 0.001), AP (*r* = −0.383, *p* < 0.001), and PR (*r* = −0.404, *p* < 0.001) were significantly negatively correlated with TA. Furthermore, AS (*r* = −0.446, *p* < 0.001) and MP (*r* = −0.099, *p* < 0.001) were negatively correlated with PR, whereas SS (*r* = 0.592, *p* < 0.001) and AP (*r* = 0.401, *p* < 0.001) showed significant positive correlations with PR.

**Table 2:** Descriptive analysis of the main variables and correlations between the variables.

Variables	1.AS	2.SS	3.AP	4.MP	5.PR	6.TA
Gender	−0.083^**^	0.128^***^	0.005	0.055	0.043	0.024
Age	−0.086^**^	−0.053	0.012	−0.060^*^	0.054	−0.104^**^
Grade	−0.049	−0.071^*^	−0.033	−0.068^*^	−0.029	−0.048
Major	0.087^**^	−0.027	0.017	0.008	−0.082^**^	0.058
2.	−0.387^***^	1				
3.	−0.220^***^	0.366^***^	1			
4.	0.402^***^	−0.052	0.061^*^	1		
5.	−0.446^***^	0.592^***^	0.401^***^	−0.099^***^	1	
6.	0.511^***^	−0.322^***^	−0.383^***^	0.321^***^	−0.404^***^	1
*K*	2.654	0.459	0.724	0.862	0.548	0.218
*S*	−1.091	0.134	−0.146	−0.157	0.141	−0.182
*M*	2.933	4.454	3.099	3.065	3.284	18.400
SD	0.456	1.072	0.726	0.650	0.685	5.953

N = 1,067.

^*^p <0.05; ^**^p <0.01; ^***^p <0.001.

AS, Academic Stress; SS, Social Support; AP, Adaptive Perfectionism; MP, Maladaptive Perfectionism; PR, Psychological Resilience; TA, Test Anxiety.

### 4.2 Common method bias

Given that all variables were measured through participants' self-assessments, the study accounted for the risk of method variance by employing Harman's single-factor approach. The unrotated factor solution identified 20 distinct components (eigenvalues > 1), with the leading factor explaining just 14.622% of the total variance. Since this value is considerably lower than the 50% cutoff suggested by Podsakoff and Organ (1986), the findings suggest that bias from a single measurement source was not a substantial threat in the present analysis.

### 4.3 Confirmatory factor analysis

AMOS was used in this study to assess the fit and structural validity of the proposed research models. As shown in Table 3, the χ^2^/*df* values for Model A, Model B, Model C, and Model D were 2.619, 2.305, 2.437, and 2.462, respectively—all below the recommended threshold of three, indicating an acceptable model fit. Additionally, all four models had RMSEA values below 0.05, suggesting minimal model error and meeting the criteria for a good fit. The structural equation modeling fit indices further indicated that TLI, CFI, GFI, and AGFI were all above 0.70, demonstrating that the models achieved an acceptable level of fit (Hair et al., 2010). Although some indicators did not exceed the ideal threshold of 0.90, the overall fit results provide sufficient support for subsequent path analysis and interpretation.

**Table 3:** Fitting index of structural equation model.

Model	χ^2^*/df*	RMSEA	TLI	CFI	GFI	AGFI
Model A (*X* = AS)	2.619	0.039	0.725	0.735	0.832	0.821
Model B (*X* = SS)	2.305	0.035	0.840	0.846	0.870	0.860
Model C (*X* = AP)	2.437	0.037	0.806	0.814	0.879	0.869
Model D (*X* = MP)	2.462	0.037	0.798	0.807	0.878	0.868

N = 1,067.

AS, Academic Stress; SS, Social Support; AP, Adaptive Perfectionism; MP, Maladaptive Perfectionism.

### 4.4 Mediation effect analysis

**Figure 2:**
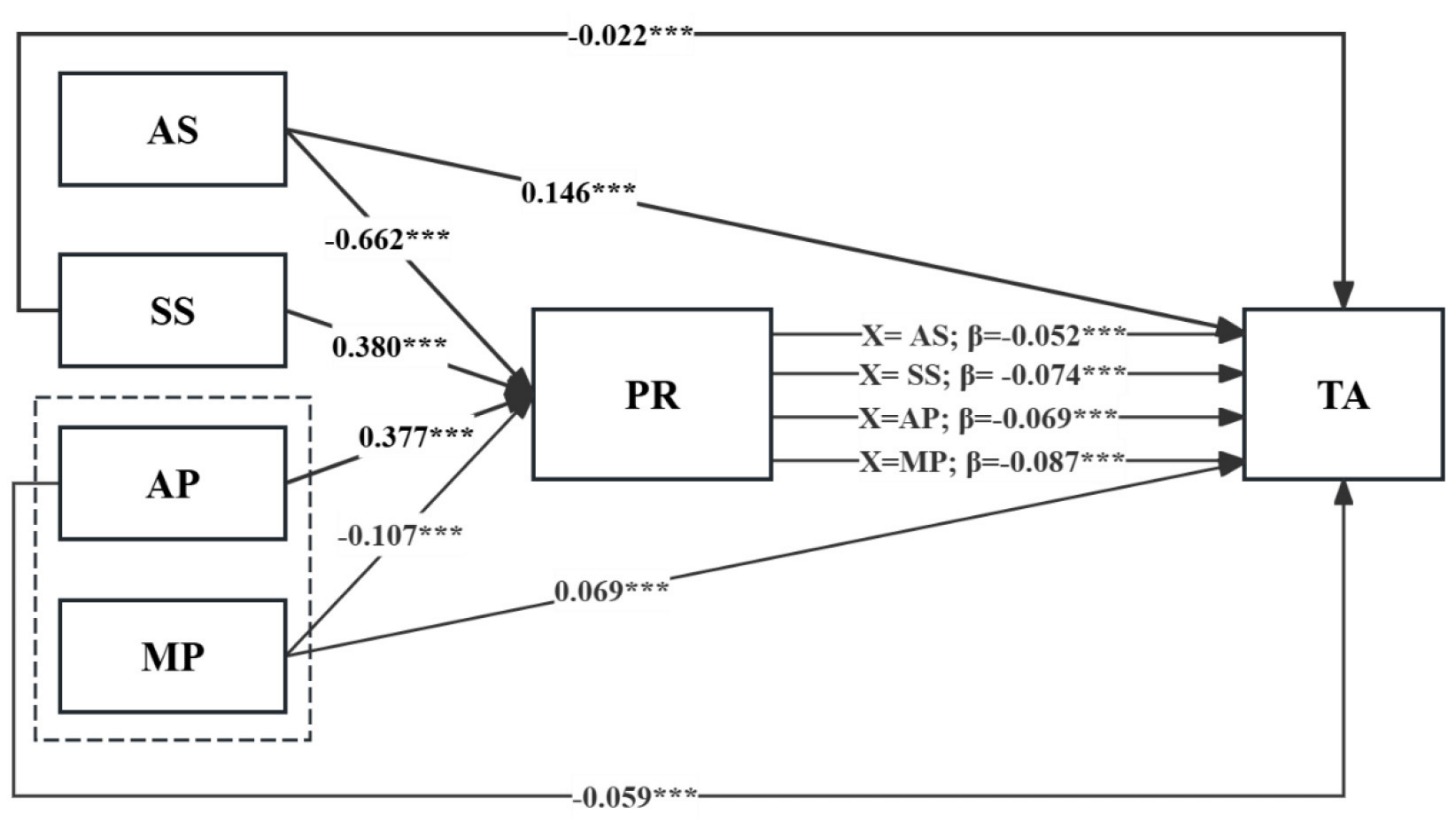
Intermediary model. ***p < 0.001; AS, Academic Stress; SS, Social Support; AP, Adaptive Perfectionism; MP, Maladaptive Perfectionism; PR, Psychological Resilience; TA, Test Anxiety.

The correlation analysis results met the statistical requirements for conducting mediation analysis. This study used Model 4 of the PROCESS macro in SPSS 26.0, with the bias-corrected percentile Bootstrap method and 5,000 bootstrap samples, to assess the effects of AS, SS, AP, and MP on TA among medical students and test the mediating role of PR. The regression analysis results are shown in Table 4 and Figure 2.

**Table 4:** Regression analysis between the variables.

Outcome variable	Predictor variable	β	SE	*T*	Bootstrap 95% CI		*R* ^2^	*F*
					LLCI	ULCI		
AS→PR→TA	
PR	AS	−0.662^***^	0.042	−15.920	−0.723	−0.580	0.206	55.195
TA	AS	0.146^***^	0.010	14.354	0.126	0.166	0.307	78.096
TA	PR	−0.052^***^	0.007	−7.651	−0.065	−0.038		
SS→PR→TA	
PR	SS	0.380^***^	0.016	24.062	0.349	0.411	0.364	121.421
TA	SS	−0.022^***^	0.005	−4.210	−0.032	−0.012	0.185	40.202
TA	PR	−0.074^***^	0.008	−9.024	−0.090	−0.058		
AP→PR→TA	
PR	AP	0.377^***^	0.026	14.315	0.325	0.429	0.176	45.322
TA	AP	−0.059^***^	0.007	−9.065	−0.072	−0.046	0.231	53.169
TA	PR	−0.069^***^	0.007	−9.858	−0.082	−0.055		
MP→PR→TA	
PR	MP	−0.107^***^	0.032	−3.349	−0.170	−0.044	0.027	5.920
TA	MP	0.069^***^	0.007	10.388	0.056	0.082	0.248	58.348
TA	PR	−0.087^***^	0.006	−13.776	−0.100	−0.075		

N = 1,067; ^***^p <0.001.

AS, Academic Stress; SS, Social Support; AP, Adaptive Perfectionism; MP, Maladaptive Perfectionism; PR, Psychological Resilience; TA, Test Anxiety.

In the mediation path from AS to TA, AS significantly negatively predicted PR (β = −0.662, *p* < 0.001), significantly positively predicted TA (β = 0.146, *p* < 0.001), and PR significantly negatively predicted TA (β = −0.052, *p* < 0.001). In the mediation path from SS to TA, SS significantly positively predicted PR (β = 0.380, *p* < 0.001), significantly negatively predicted TA (β = −0.022, *p* < 0.001), and PR significantly negatively predicted TA (β = −0.074, *p* < 0.001). In the mediation path from AP to TA, AP significantly positively predicted PR (β = 0.377, *p* < 0.001), significantly negatively predicted TA (β = −0.059, *p* < 0.001), and PR significantly negatively predicted TA (β = −0.069, *p* < 0.001). In the mediation path from MP to TA, MP significantly negatively predicted PR (β = −0.107, *p* < 0.001), significantly positively predicted TA (β = 0.069, *p* < 0.001), and PR significantly negatively predicted TA (β = −0.087, *p* < 0.001). Therefore, Hypotheses H1, H2, H3, and H4 were supported.

The results of the mediation analysis (as shown in Table 5) indicate that the total effect of AS on TA was 0.18, with a direct effect of 0.146. When PR was included as a mediator, the indirect effect was 0.034, accounting for 18.889% of the total effect. For SS, the total effect on TA was −0.050, with a direct effect of −0.022. The indirect effect via PR was −0.028, accounting for 56% of the total effect. The total effect of AP on TA was −0.085, with a direct effect of −0.059, and an indirect effect through PR of −0.026, representing 30.588% of the total effect. For MP, the total effect on TA was 0.079, with a direct effect of 0.069, and an indirect effect of 0.010 via PR, accounting for 12.658% of the total effect. Therefore, Hypotheses H5, H6, H7, and H8 were supported.

**Table 5:** Analysis of intermediation effects.

Effect	Path	β	Percentage	Bootstrap 95% CI	
				LLCI	ULCI
*X* = AS	
Total effect	AS → TA	0.18	100%	0.162	0.199
Direct effect	AS → TA	0.146	81.111%	0.126	0.166
Indirect effect	AS → PR → TA	0.034	18.889%	0.023	0.046
*X* = SS	
Total effect	SS → TA	−0.05	100%	−0.059	−0.042
Direct effect	SS → TA	−0.022	44%	−0.032	−0.012
Indirect effect	SS → PR → TA	−0.028	56%	−0.037	−0.020
*X* = AP	
Total effect	AP → TA	−0.085	100%	−0.097	−0.073
Direct effect	AP → TA	−0.059	69.412%	−0.072	−0.046
Indirect effect	AP → PR → TA	−0.026	30.588%	−0.035	−0.018
*X* = MP	
Total effect	MP → TA	0.079	100%	0.064	0.093
Direct effect	MP → TA	0.069	87.342%	0.056	0.082
Indirect effect	MP → PR → TA	0.01	12.658%	0.002	0.017

N = 1,067.

AS, Academic Stress; SS, Social Support; AP, Adaptive Perfectionism; MP, Maladaptive Perfectionism; PR, Psychological Resilience; TA, Test Anxiety.

## 5 Discussion

Focusing on the psychological mechanisms underlying TA in medical students, this study assessed the impact of AS, SS, and perfectionistic traits, while also probing the intermediary role of PR. The proposed conceptual pathways were empirically tested through statistical analyses performed in SPSS 26.0. The following discussion elaborates on the findings in relation to each hypothesis.

The current findings identified a significant positive association between AS and TA in medical students. These outcomes were consistent with the research of Zheng et al. (2023), who found that university students experiencing higher levels of AS tended to report lower emotional self-efficacy, which was linked to higher TA. According to the Control-Value Theory (Pekrun, 2006), when facing heavy academic demands and frequent examinations, medical students might have perceive a lower sense of control due to both internal and external pressures. Simultaneously, factors like academic expectations and peer competition may lead students to place more importance on their final exams. TA, as an outcome-focused negative activating emotion, can emerge in situations characterized by low perceived control and high perceived value, thereby contributing to heightened TA levels among medical students (Pekrun et al., 2007). Accordingly, elevated AS was associated with higher levels of TA among medical students.

Results also revealed that increased SS was associated with reduced TA, which aligns with findings from Tsegay et al. (2019), whose research demonstrated that inadequate SS was linked to heightened anxiety during examinations. Drawing upon Control-Value Theory (Pekrun, 2006) and the stress-buffering model (Cohen and Wills, 1985), emotional, instrumental, and informational support from friends, family, and instructors were seen as valuable psychological resources. Such support may enhance students' self-confidence and increase their perceived control over examinations (Cobb, 1976). Moreover, positive SS can help students moderate their expectations regarding exam outcomes, thereby reducing the perceived value attributed to performance results (Poots and Cassidy, 2020). As such, medical students who perceived adequate SS tend to exhibit reduced levels of TA.

This study also confirmed that AP was negatively associated with TA, while MP was positively associated with TA among medical students. These results are consistent with the findings of Vanstone and Hicks (2019), who demonstrated that both types of perfectionism influence TA through the mediating effect of avoidant emotion-focused coping. According to Control-Value Theory (Pekrun, 2006) and Perfectionism Theory (Hewitt and Flett, 1991), individuals exhibiting AP typically approach academic tasks with a confident mindset, experience a heightened sense of agency during evaluations, and show tolerance for imperfection even as they pursue ambitious goals. They are more likely to evaluate exam outcomes more rationally, which leads to relatively lower levels of TA (Madigan, 2019). In contrast, students high in MP often struggle to tolerate failure, tend to be overly self-critical, and feel a diminished sense of control during exams. They may tend to magnify their mistakes and hold extreme beliefs about the value of exam outcomes, which can contribute to higher levels of TA (Weiner and Carton, 2012). Thus, medical students with stronger AP are associated with lower TA, whereas those with stronger MP were more likely to report elevated anxiety levels.

Findings indicated that PR functioned as an intermediary in the link between AS and TA. Specifically, when medical students perceived a high level of AS, their level of PR tended to decrease in exam-related situations, which may led to higher levels of TA. Mediation analysis revealed that the indirect effect of PR accounted for 18.889% of the total effect between AS and TA. Similar outcomes were reported in the studies conducted by Berdida (2023) and Trigueros et al. (2020), reinforcing the present finding. According to Resilience Theory (Masten, 2001) and the stress-vulnerability model (Zubin and Spring, 1977), when people encounter external pressures without the support of effective internal coping capacities, they may become increasingly vulnerable to negative psychological consequences such as TA. As a key protective mechanism, PR enables medical students to maintain mental health when facing adversity. Therefore, when medical students are under pressure from academic performance, workload, and professional development, those with low resilience are more prone to emotional breakdowns and heightened anxiety, whereas those with strong PR are more likely to maintain cognitive and emotional stability, regulate negative emotions, and therefore experience lower levels of TA (Troy et al., 2023). In this context, PR not only serves as a structural mediator in the pathway from AS to TA, but also plays a functional buffering and transforming role. These results highlight PR as a core psychological factor through which AS is associated with students' experience of TA. This mediation mechanism illustrated how AS may exacerbate TA by depleting individual psychological resources and offered both a theoretical foundation and practical target for psychological interventions and stress management programs in educational settings.

This study found that PR mediates the relationship between SS and TA. Specifically, when medical students perceived adequate SS, their level of PR tended to be enhanced during exam periods, which may help alleviate TA. The mediation results showed that PR explained 56% of the overall association between SS and TA. This finding is consistent with Liu and Cao (2022), who found that SS strengthened PR in medical students, helping to alleviate the detrimental effects of academic burnout on their academic engagement. According to the compensatory model of Resilience Theory (Fergus Fergus and Zimmerman, 2005) and the stress-buffering model (Cohen and Wills, 1985), SS functions as an external resource that supports resilience. It strengthens individuals' coping capacity by enhancing perceived competence, self-efficacy, and emotional regulation (Shengyao et al., 2024), thereby improving their PR. This interactive mechanism between external resources and internal strengths enables individuals to withstand external threats, reduce the psychological stress caused by exams, and lower levels of TA. Therefore, in high-stress examination contexts, when medical students received sufficient SS, they were more likely to develop stronger PR, which may help effectively mitigate TA.

Findings indicate that PR acted as a key mechanism through which both forms of perfectionism—adaptive and maladaptive—influenced TA in medical students. Specifically, students with stronger AP tended to exhibit higher levels of PR, which may help lower their TA. In contrast, a stronger tendency toward MP may reduce PR, thereby increasing TA. Mediation analysis revealed that PR accounted for 30.588% of the total effect between AP and TA, and 12.658% of the total effect between MP and TA. According to the challenge stressor model (Lepine et al., 2005) and Resilience Theory (Masten, 2001), individuals with AP often possess strong goal orientation, persistence, and self-efficacy, and are more capable of viewing failure and challenges rationally. Such personal attributes can facilitate the strengthening of one's capacity to adapt and recover from adversity. Research has shown that such individuals are often more flexible in emotional regulation and proactive in coping with stress, thereby indirectly reducing TA (Tugade and Fredrickson, 2004). These positive traits align with the “positive trait–adaptive outcome” pathway in Resilience Theory and represent a key internal component of resilience (Ong et al., 2006). On the other hand, MP reflects what Resilience Theory refers to as a “risk trait–maladaptive pathway.” When individuals consistently hold negative cognitions about failure—characterized by heightened self-blame and an intense fear of errors—their self-efficacy and coping capabilities may deteriorate, leading to reduced PR and entrenchment in a cycle of negative emotions (Oshio et al., 2018). For these individuals, the recovery of resilience was influenced by both external support and the need to modify their internal cognitive and emotional patterns. Although adaptive and MP shared surface similarities, they differed fundamentally in function. Adaptive perfectionism exerted a distinctly positive influence by enhancing PR, which helped to alleviate TA among medical students.

## 6 Implications

### 6.1 Theoretical implications

Firstly, this research innovatively integrates Control-Value Theory and Resilience Theory to construct a multilevel mediation model, offering a novel perspective for understanding TA among medical students. Unlike previous studies that typically relied on a single theoretical framework, this study incorporates the unique psychological characteristics of medical students, integrating multiple variables such as situational factors, personality traits, and environmental support. It explores the mediating role of PR in the relationship between AS and TA. The integration of these diverse theoretical frameworks not only enhances the explanatory power of the theory but also broadens the developmental path of psychological models.

Secondly, this study departs from traditional linear “stress-anxiety” models by proposing a multivariable path model. The model considers the interaction of multiple factors, including AS, AP, MP, and SS, and reveals the role of PR as a mediating variable. The innovation of this model lies in its focus not only on cognitive appraisals involved in the formation of anxiety but also on how positive psychological resources can buffer against negative emotional experiences, thus transitioning from a “risk-generation” model to a “resource-conservation” model. It emphasizes the essential function of individual psychological resources in coping with stress.

Additionally, the study's detailed analysis of perfectionism and TA provides new insights into Resilience Theory. It was found that adaptive perfectionism alleviates academic anxiety indirectly by enhancing psychological resilience, whereas maladaptive perfectionism exacerbates anxiety by undermining resilience. This finding enriches the understanding of the relationship between perfectionism and mental health, offering new theoretical support for the application of Resilience Theory in future studies across different populations.

### 6.2 Practical implications

This research offers valuable guidance for medical students. The findings indicate that AS and MP are linked to TA, while SS, AP, and PR function as protective factors that may buffer against anxiety. The results help students understand the psychological dynamics behind their emotional states and how different forms of perfectionism may influence their stress and coping patterns. By engaging in self-regulation techniques such as self-acceptance, mindfulness training, and positive emotion regulation, students can gradually strengthen their PR, enabling them to manage exam-related stress more effectively. Additionally, students should be encouraged to actively seek SS by maintaining communication with family, friends, and mentors to build a supportive network.

This study also offers practical guidance for university instructors. The results highlight that exam stress and perfectionist tendencies contribute to TA, which are influenced by teaching methods and assessment practices. Educators should plan course pacing and exam scheduling carefully to avoid overloading students. Instructors should also be alert to perfectionist tendencies and anxiety symptoms among students, offering timely emotional support. Fostering positive teacher–student relationships and a constructive classroom environment can enhance students' sense of control, indirectly improving their PR.

At the institutional level, this study provides insights for improving psychological support systems. These outcomes emphasize the role of SS and PR in mitigating students' anxiety, suggesting that institutions develop well-structured support frameworks tailored to student needs. Institutions could establish comprehensive mental health services, such as psychological assessments, resilience-building workshops, and counseling programs during exam periods. Additionally, a three-tier collaborative support system involving counselors, mental health professionals, and academic advisors could be developed. Institutions may also explore reforming assessment systems to incorporate diverse evaluation methods, reducing reliance on high-stakes exams. These initiatives help lower TA and support the development of resilient, capable medical professionals.

## 7 Limitations and future research

This research utilized a cross-sectional survey approach, which, although useful for identifying associations between variables, restricts the interpretation of long-term causal effects. For instance, it remains uncertain whether TA may in turn influence PR or perceived SS—a possibility that warrants further investigation. Clarifying the directional relationships between these factors may require future research to utilize time-series or follow-up designs that capture students' mental state fluctuations during different stages of assessment. Alternatively, experimental approaches—like resilience-focused interventions—could be employed to directly test their effectiveness in reducing TA. Furthermore, all variables within the present study were measured through the use of self-report scales, which may be influenced by extraneous factors such as social expectations and the emotional state of the subjects, thus leading to a certain degree of response bias. It is also possible that subjective differences in how participants understood constructs such as perfectionism and SS affected the results. For a deeper exploration of how students navigate examination pressure and interpersonal dynamics, subsequent studies might adopt qualitative techniques like narrative interviews, free-text responses, or longitudinal self-report diaries to enrich the interpretive depth. These approaches may yield richer psychological insights and help compensate for the limitations of purely quantitative research.

## 8 Conclusion

In undergraduate medical education, students are primarily assessed through examinations, coupled with heavy academic workloads and the pressures of postgraduate entrance exams and employment, making them particularly susceptible to high levels of TA. This study explored the psychological pathways through which AS, SS, and perfectionistic traits influence TA, focusing on the mediating role of PR. The findings showed that AS and MP were positively associated with TA, while SS and AP were negatively associated with TA. PR acted as a mediator in all pathways. By integrating Control-Value Theory and Resilience Theory, this study provides a comprehensive approach to understanding TA, offering new insights into psychological research in medical education. These outcomes have practical implications for medical education, supporting evidence-based strategies to alleviate exam-related stress and promote emotional resilience and academic success. However, the use of a cross-sectional design limited the ability to establish causal relationships among the core variables. Longitudinal studies are needed to explore developmental trends and directional influences. Additionally, the reliance on self-reported data may introduce biases from social desirability or subjective interpretation. Future research could enhance methodological rigor by incorporating mixed-method approaches, such as interviews or third-party evaluations, to provide more reliable insights.

## Data Availability

The datasets presented in this study can be found in online repositories. The names of the repository/repositories and accession number(s) can be found in the article/supplementary material.
